# Role of Sex Hormones on Brain Mitochondrial Function, with Special Reference to Aging and Neurodegenerative Diseases

**DOI:** 10.3389/fnagi.2017.00406

**Published:** 2017-12-07

**Authors:** Pauline Gaignard, Philippe Liere, Patrice Thérond, Michael Schumacher, Abdelhamid Slama, Rachida Guennoun

**Affiliations:** ^1^U1195 Inserm and University Paris-Sud and University Paris-Saclay, Le Kremlin-Bicêtre, France; ^2^Biochemistry Laboratory, Bicêtre Hospital, Assistance Publique-Hôpitaux de Paris, Le Kremlin-Bicêtre, France

**Keywords:** aging, progesterone, estrogens, oxidative phosphorylation, oxidative stress, sex differences, neurodegenerative diseases

## Abstract

The mitochondria have a fundamental role in both cellular energy supply and oxidative stress regulation and are target of the effects of sex steroids, particularly the neuroprotective ones. Aging is associated with a decline in the levels of different steroid hormones, and this decrease may underline some neural dysfunctions. Besides, modifications in mitochondrial functions associated with aging processes are also well documented. In this review, we will discuss studies that describe the modifications of brain mitochondrial function and of steroid levels associated with physiological aging and with neurodegenerative diseases. A special emphasis will be placed on describing and discussing our recent findings concerning the concomitant study of mitochondrial function (oxidative phosphorylation, oxidative stress) and brain steroid levels in both young (3-month-old) and aged (20-month-old) male and female mice.

## Introduction

The cross-talk between mitochondria and sex steroids plays a major role in the brain. Indeed, sex steroids influence numerous functions of mitochondria: energy production, oxidative stress regulation, calcium homeostasis, cell proliferation or apoptosis (Nilsen and Diaz Brinton, 2003; Chen et al., 2009a,b; Sayeed et al., 2009; Gaignard et al., 2017). In addition, because mitochondria are also the site of the first step of steroidogenesis, dysfunctions of mitochondria may impact on steroidogenesis. Since sex steroids decrease and mitochondrial alterations are known to be implicated in aging, understanding the relationship between both is a key to explore normal and pathological brain aging. This review will focus on two intricate mitochondrial functions: the energy production by oxidative phosphorylation and the regulation of oxidative stress. After a brief summary of age-associated mitochondrial dysfunction in brain and the decrease of brain steroids, we discuss the regulation of mitochondrial metabolism by sex steroids in the context of aging. We then expose the current knowledge about sexual dimorphism in mitochondrial function during normal brain aging and in neurodegenerative diseases, and we emphasize the role of sex steroids on it.

## The Decline of Mitochondrial Function during Aging

**Figure 1:**
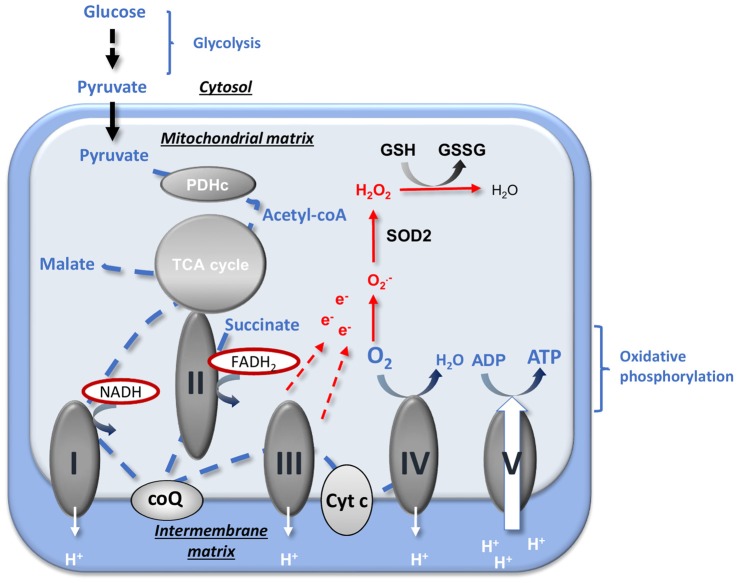
Mitochondrial energy production and reactive oxygen species (ROS) regulation. Pyruvate from an aerobic glycolysis is converted by pyruvate dehydrogenase complex (PDHc) into acetyl-coA that enters into the tricarboxylic acid (TCA) cycle. The reducing equivalents NADH and FADH_2_ produced are used by the respiratory chain (RC) complexes (complexes I, II, III and IV) to reduce O_2_ (« respiration ») and to create the proton gradient necessary to ATP production by complex V. The oxidative phosphorylation is the coupling between ADP phosphorylation and the electron transport. The production of anion superoxide 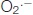 is inherent to mitochondrial respiration because of the proximity between free electrons and O_2_. ROS detoxification is provided by the SOD2 that catalyzes the dismutation of 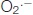 into H_2_O_2_ and by GSH that allows the reduction of H_2_O_2_ into H_2_O. CoQ, ubiquinone; Cyt c, cytochrome c; e^−^, electron; GSH, reduced glutathione; GSSG, oxidized glutathione; 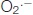, anion superoxide; PDHc, pyruvate dehydrogenase complex; SOD, superoxide dismutase; TCA, tricarboxylic acid. Adapted from Gaignard et al. (2017).

Energy production and oxidative stress regulation by mitochondria are critical for cell life and particularly in the brain that has both a high metabolic rate and an increased sensitivity to oxidative damages (Kann et al., 2007). The mitochondrial ATP production from pyruvate coming from glycolysis requires three principal enzymatic systems (Figure 1): the pyruvate dehydrogenase complex (PDHc), composed of three subunits E1, E2 and E3 that convert pyruvate to acetyl-coA; the tricarboxylic acid (TCA) cycle that produces reduced co-enzymes (reduced nicotinamide adenine nucleotide NADH and reduced flavin adenine dinucleotide FADH_2_) and the respiratory chain (RC) that finally produces ATP. The RC is composed of five complexes, the first four complexes (complexes I, II, III and IV) form the electron transfer chain (ETC) that creates an electrochemical gradient and reduces O_2_ into H_2_O (“respiration”). The complex V then catalyzes the phosphorylation of ADP into ATP using the proton motive force generated by the ETC. The coupling between the electron transport and the ADP phosphorylation is called “oxidative phosphorylation”.

In addition to energy production, mitochondria are a major cellular regulators of oxidative stress by producing reactive oxygen species (ROS) and harboring powerful antioxidant systems (Wüllner et al., 1999; Murphy, 2009; Fukui and Zhu, 2010). ROS generation and mitochondrial respiration are intrinsically linked. The close proximity between high concentrations of O_2_ and of electrons promotes the production of superoxide anions (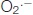), especially when the ETC is accelerated or, on the contrary, is slowed down (Murphy, 2009). The regulation of oxidative stress is principally mediated by the mitochondrial superoxide dismutase SOD2 (or MnSOD) that catalyzes the dismutation of superoxide anions to H_2_O_2_ and by the reduced glutathione (GSH) pool that allows the detoxification of H_2_O_2_ into H_2_O (Figure 1). The maintenance of the GSH pool and of the ratio between reduced and oxidized glutathione forms (GSH/GSSG) is especially crucial for the brain, as the activity of catalase, the other enzyme that detoxifies H_2_O_2_, is low in this tissue (Kudin et al., 2012).

In 1956, Harman (1956) exposed the “free radical theory of aging” suggesting that oxidative damages accumulate with age and are responsible for aging. Based on the central role of mitochondria in ROS production and detoxification, this theory has evolved into the “mitochondrial aging theory”: oxidative damages induced by free radicals on mitochondrial DNA (mtDNA) particularly cause mitochondrial impairments that enhance ROS production in a vicious circle, finally leading to cellular failure (Harman, 1972). Since several decades, the age-induced mitochondrial alterations have been largely studied and numerous experimental evidence support Harman’s theory. However, the direct link between free radicals, mtDNA mutations and cellular alterations during aging has become controversial, and it has been proposed that mitochondrial ROS are involved in aging process by their roles in cellular signaling rather than by their altering effects (Stuart et al., 2014).

With regard to brain aging, investigating the “mitochondrial aging theory” is particularly relevant. The brain is highly vulnerable to oxidative damages because of the scarcity of antioxidant defense systems in this tissue (Poon et al., 2006; Kann et al., 2007). The accumulation of oxidative damages in brain mitochondrial proteins, lipids and nucleotides has been demonstrated in numerous studies (reviewed in Chakrabarti et al., 2011). Thus, it has been reported that the activity of the TCA cycle enzyme α-keto-glutarate dehydrogenase (αKGDH) was diminished under pro-oxidative conditions (Tretter and Adam-Vizi, 2005); the cardiolipin, a specific mitochondrial membrane lipid, was subjected to peroxidation during aging and the peroxidized cardiolipin form inhibited complex IV activity in brain, which could potentially disrupt RC complexes interactions (Petrosillo et al., 2008, 2013; Lenaz and Genova, 2010). In pigmented neurons of the human substantia nigra, the accumulation of mtDNA deletions and the decrease in complex IV activity were correlated (Kraytsberg et al., 2006). Nevertheless, the aging consequences on RC efficacy are much less established: the studies reported either a decline or no change on RC function (reviewed in Gilmer et al., 2010; Chakrabarti et al., 2011). Methodological and experimental points could explain some of discrepancies between the age-effect studies. The age selected for the “aged” rodent group is not always similar (either 12- or 24-month-old generally) and has to be in concordance with the lifespan specificities of the species and strains studied (Turturro et al., 1999); the methods exploring RC function do not reach the same conclusions according to the energetic substrate used to measure oxygen consumption or the exhaustiveness in the analysis of enzymatic activities. Importantly, the aging-effect observed in mitochondria isolated from whole brain could differ from those observed in mitochondria isolated from brain sub-regions, since different patterns of age-related changes on RC function were described between brain areas. For example, mitochondria from hippocampus of male rats presented higher age-induced changes in mitochondrial bioenergetics than those isolated from various other areas (Pandya et al., 2016). In accordance, the rate of mtDNA mutations and activation of base excision repair pathways were not affected by aging in the same way according to brain sub-regions (McInerny et al., 2009; Gredilla et al., 2010). At the cellular level, differences between synaptic and non-synaptic mitochondria were reported; with a decrease of complex I activity with age in non-synaptic mitochondria but not in synaptic ones (Ferrándiz et al., 1994).

In humans, the effect of aging on mitochondrial metabolism has been mainly analyzed in skeletal muscle and less in brain. Two studies reported a decrease in complex IV activity or protein quantity in different brain sub-regions (Ojaimi et al., 1999; Cottrell et al., 2001), and another reported an increase in oxidative damages and a decreasing trend in complex I activity in frontal cortex and in hippocampus (Venkateshappa et al., 2012).

## The Decline of Brain Steroid Levels during Aging

The decrease in the peripheral synthesis of sex steroids is a major feature of aging, with a more drastic drop in women at the time of menopause when compared to men. Of note, the age-induced changes in sex steroid levels observed in blood may differ from those observed in brain, since the brain pool of sex steroids depends both on endocrine gland production and on the local synthesis of neurosteroids (Schumacher et al., 2003). Nevertheless, the brain also suffers from a decline in sex steroids. We have recently reported the profile of progesterone and its metabolites in the brain of aged male and female mice and showed an overall age-induced decrease (Gaignard et al., 2015). In the limbic region, Caruso et al. (2013) described also a decrease of 17β-estradiol, progesterone, testosterone and some metabolites by comparing 7- and 24-month-old male mice. Due to evident technical limitations, the knowledge of age-dependent changes in human brain levels of sex steroids is still fragmented. A recent RIA study reported that androgen but not estrogen levels declined in mid-frontal gyrus of men from 50 years to 97 years, whereas no correlation between androgen or estrogen brain levels and age were found in aged women (Rosario et al., 2011). Complementary studies are now needed in younger populations but also in various brain regions, since changes in steroid profiles differ between brain areas (Weill-Engerer et al., 2002).

**Figure 2:**
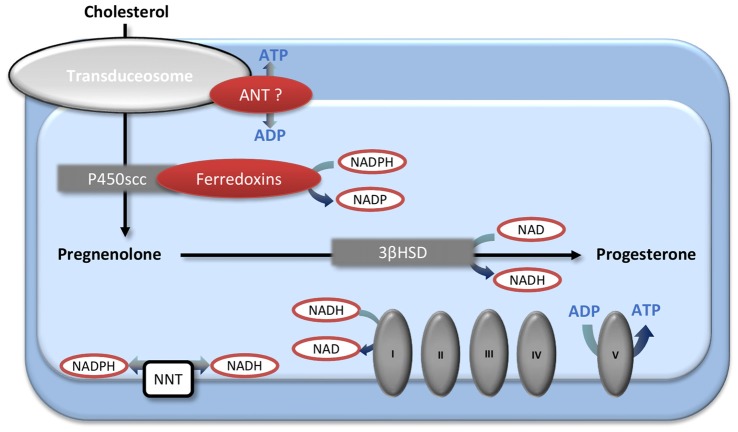
The mitochondrial first steps of steroidogenesis and their interactions with energetic metabolism. Cholesterol enters into the mitochondria by the transduceosome and is converted into pregnenolone by the P450scc. The 3βHSD, located both in mitochondria and endoplasmic reticulum, produces progesterone from pregnenolone. Mitochondrial energetic metabolism and steroidogenesis could interact by several ways. The ADP/ATP transporter adenine nucleotide transporter (ANT) may be a part of the transduceosome; the P450scc-associated ferredoxins are regulated by the NADPH/NADP ratio that is linked with the NADH/NAD ratio by the transhydrogenase NNT; NAD is a co-factor of 3βHSD. 3βHSD, 3β-hydroxysteroid dehydrogenase; ANT, adenine nucleotide transporter; NNT, nicotinamide nucleotide transhydrogenase; P450scc, cholesterol side-chain cleavage enzyme cytochrome P450.

Interestingly, the age-induced decline in mitochondrial function could modify sex steroid levels on its own. Thus, mitochondria are not only the targets of sex steroid actions but also the site of the initial steps of steroidogenesis. Cholesterol, the precursor of all steroid hormones, enters the mitochondria via the transduceosome complex where it is converted into pregnenolone by the mitochondrial cholesterol side-chain cleavage enzyme cytochrome P450 (P450scc). Then, the 3β-hydroxysteroid dehydrogenase (3βHSD) produces progesterone from pregnenolone. The 3βHSD is located both in mitochondria and in the endoplasmic reticulum (Miller, 2013). Some data suggest a potential regulation between mitochondrial metabolism and steroidogenesis (Figure 2). The exact composition and the functioning of the transduceosome are not fully elucidated (Papadopoulos et al., 2015; Selvaraj and Stocco, 2015), but it has been suggested that the adenine nucleotide transporter (ANT) may be part of it (Midzak et al., 2011). Yet, ANT is also responsible for the ADP/ATP transfer through the mitochondrial membrane. Second, the ferredoxins associated with cytochrome P450scc are regulated by the NADP/NADPH ratio (reviewed in Miller, 2005). NADP/NADPH ratio is the phosphorylated counterpart of the NAD/NADH ratio which is mainly regulated by the TCA cycle and RC in mitochondria. The NAD(H) and the NADP(H) pools are compartmentalized in cells, but it has been demonstrated that they are connected by the nicotinamide nucleotide transhydrogenase (NNT; Fisher-Wellman et al., 2015). In addition, the 3βHSD utilizes NAD as a co-factor (Chapman et al., 2005). Finally, as hydroxysteroid dehydrogenases enzymes use the nicotinamide cofactors to interconvert steroid hormones, and as their activities depend mainly on cofactors abundance, levels of steroids may be modulated by the levels of these cofactors and consequently by the redox state of the cells (Agarwal and Auchus, 2005). In light of this information, further specific investigations must determine if alterations in brain mitochondrial energetic function may impact neurosteroidogenesis. This would be another mechanism leading to age-induced brain steroids decrease, in addition to the decline of the peripheral synthesis and to the decrease of expression of the brain enzymes involved in *in situ* production of steroids (Velarde, 2014; Rossetti et al., 2015).

## The Regulation of Mitochondrial Metabolism by Sex Steroids and The Particular Context of Aging

Since few years, there is a growing interest in the effects of sex steroids on brain mitochondrial metabolism. While the mitochondrial effects of testosterone are not well documented, several pharmacological studies have shown that exogenous administration of 17β-estradiol and/or progesterone increases RC function and decreases oxidative stress in brain mitochondria (reviewed in Chen et al., 2009a,b; Rettberg et al., 2014; Gaignard et al., 2017). It has also been demonstrated that these mitochondrial effects contribute to the neuroprotective properties of sex steroids after brain injury (Robertson et al., 2006; Guo et al., 2012; Robertson and Saraswati, 2015; Webster et al., 2015; Gaignard et al., 2016; Yousuf et al., 2016; Andrabi et al., 2017).

**Figure 3:**
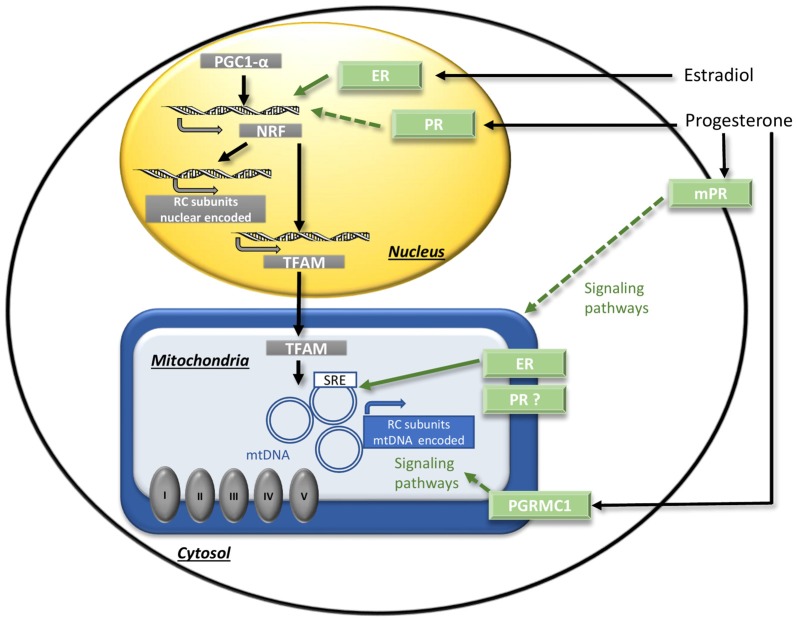
The regulation of mitochondrial function by sex steroids. The binding of estradiol to the nuclear receptors ER activates transcriptional factors (NRF/PGC1-α) that induce the expression of nuclear DNA-encoded RC subunits and of transcription factor A (TFAM). TFAM induces the expression of mitochondrial DNA (mtDNA)-encoded RC subunits. Direct transcriptional effects on mtDNA mediated by mitochondrial ER receptors could also occur. Concerning progesterone, the potential mechanisms have to be investigated. Putative signaling effects *via* NRF/PGC1-α activation by nuclear PR, or *via* membrane receptors mPR or PGRMC1 are represented by dotted arrows. ER, estrogen nuclear receptors; mPRs, progesterone membrane receptors; mtDNA, mitochondrial DNA; NRF, nuclear respiratory factor; PGC1-α, peroxisome proliferator-activated receptor gamma co-activator 1 α; PGRMC1, progesterone receptor membrane component 1; PR, progesterone nuclear receptor; RC, respiratory chain; SRE, steroid response element; TFAM, mitochondrial transcription factor A. Adapted from Gaignard et al. (2017).

It has been shown in young or ovariectomized animals that sex steroids could regulate mitochondrial energy production by transcriptional and post-transcriptional mechanisms (Figure 3). The transcriptional regulation of RC by 17β-estradiol is the best demonstrated so far. The RC compounds are encoded by two genomes, the nuclear one and the mitochondrial one, but the last encodes a few parts (only 13 RC complexes subunits out of up to 80 identified). Besides the rest of RC compounds, nuclear DNA (nDNA) encodes all machineries for the replication, the transcription and the translation of mtDNA. The nuclear respiratory factors 1 and 2 (NRF-1 and NRF-2) together with the coactivator peroxisome proliferator-activated receptor gamma coactivator 1 α (PGC1-α) and the mitochondrial transcription factor A (TFAM), operate the cross-talk between nuclear and mitochondrial genomes. The activation of the estrogen nuclear receptors (“classic” receptors) ERα or ERβ induces the expression of NRF-1, NRF-2, TFAM and of the nuclear-encoded RC subunits (reviewed in Chen et al., 2009b). Interestingly, some studies showed that the effects of ERα- and ERβ-agonists were slightly different and varied according to brain regions. Thus, in hippocampus of ovariectomized female rats, the ERβ-agonist diarylpropionitrile (DPN) was more efficient to enhance mitochondrial respiration and to decrease oxidative stress than the ERα-agonist propylpyrazoletriol (PPT; Irwin et al., 2012). By contrast, in primary human brain microvascular endothelial cells, the PPT addition decreased anion superoxide production while the DPN addition did not (Razmara et al., 2008). Considering that ERα and ERβ cerebral expressions are sexually dimorphic (Zhang et al., 2002; Sharma and Thakur, 2006), the estrogen effects on mitochondrial metabolism could be different in males and females. Recently, Riar et al. (2017) demonstrated a significant sex difference in the mitochondrial unfolded protein response (UPR^mt^), a transcriptional program that restore proteostasis, in the spinal cord of G93A-SOD1 mice and suggested that the sex difference could be due to a difference in ERα activation.

Whether or not progesterone and/or 5-α-dihydroprogesterone exert their effects via the nuclear progesterone receptor PR has to be investigated. Progesterone and derivates could also acts independently of PR. Recently, it has been shown that progesterone promoted a mild decoupling (i.e., the dissociation between the reduction of O_2_ by ETC and the ATP synthesis) in yeast cells, even when the yeast ortholog of PR was removed. This effect could be due to a direct effect on mitochondrial membranes or could be mediated by other receptors than PR (Stekovic et al., 2017). It has also been reported that 3α,5α-tetrahydroprogesterone (also known as allopregnanolone), a reduced derivate of progesterone, enhanced ATP levels and mitochondrial respiration, especially in a cellular model of Alzheimer’s disease (AD). The authors suggested that these effects were not mediated by GABA_A_ receptors, like most of the other effects of allopregnanolone, but rather by non-GABAergic receptors such as membrane PR (mPRs; Pang et al., 2013; Lejri et al., 2017).

In addition, number of arguments suggest that sex steroids could also exert their regulation via mitochondrial receptors. Thus, ERα and ERβ were detected in the mitochondria of neurons (Yang et al., 2004; Alvarez-Delgado et al., 2010) and they could potentially exert direct transcriptional effects of mtDNA because mtDNA harbors steroid response element (SRE) sequences (Demonacos et al., 1996). The existence of a similar mechanism for progesterone action is possible but must be demonstrated, since the reality of the mitochondrial form of PR is still debated. A splicing variant of *PR* gene was described in some tissues, but it encodes a truncated form of the receptor and its functionality is doubtful (Samalecos and Gellersen, 2008). Furthermore, the progesterone receptor membrane component 1 (PGRMC1) was detected in both membrane and mitochondrial fractions and co-localized with markers of the endoplasmic reticulum and the mitochondria (Meffre et al., 2005; Xu et al., 2011; Guennoun et al., 2015). By this way, progesterone could exert post-transcriptional regulation on mitochondria by activating signaling pathways (Figure 3).

After effects demonstrated in young animals, a challenging issue now is to determine whether steroids exert mitochondrial effects in aged individuals. In addition to a decline in steroid levels, the question arises of the persistence of the receptors and of their functionalities during aging. The interpretation of the experimental data is however delicate because of methodological pitfalls (mRNA levels do not necessary reflect protein levels, the presence of the receptor does not signify that it is fully functional…). Besides, the coexistence of several isoforms of nuclear sex steroid receptors adds even more complexity. In female mice, a study reported that the quantity of nuclear receptor ERα was increased in the anteroventral periventricular nucleus of the hypothalamus in aged females, but that the quantity of ERβ was decreased in the same region (Chakraborty and Gore, 2004). In the cortex, one study showed a decrease in both ERα and ERβ protein levels in aged female mice when compared to young females (Cai et al., 2014), but another one did not detect significant changes in ERα protein levels (Dietrich et al., 2015). Interestingly, it has been reported that the mitochondrial ERα and ERβ content did not vary between young and aged female rats in cortex, hippocampus and hypothalamus (Alvarez-Delgado et al., 2010). In male mice, no significant variations in mRNA levels of ERα and ERβ was observed between 12- and 24-month-old mice, but protein levels were not examined (Munetomo et al., 2015). Recently, the age-associated changes in ER levels and distribution were reviewed by Hara et al. (2015), underpinning that they vary across species and brain regions.

Age-associated decrease in progesterone receptor (PR) expression has been reported in the hypothalamus of male and female rhesus macaques; however their expression still responded to estradiol supplementation (Naugle et al., 2014; Eghlidi and Urbanski, 2015; Eghlidi et al., 2017). A decrease in PR mRNA expression during reproductive aging in the hypothalamus of female rats was also demonstrated (Mills et al., 2002). Analysis of estrogenic regulation of PR mRNA in hypothalamus of aged female rats showed that the induction of PR mRNA by estrogen treatment in middle-aged rats is as strong as in young rats (Funabashi et al., 2000). However, analysis by immuno-histochemistry, showed that the ability of estradiol to stimulate PR expression is attenuated with aging in female rats (Furuta et al., 2010). In the cerebral cortex of aged female mice, one study showed that PR mRNA expression declined with age, but PR protein levels were sustained (Dietrich et al., 2015). The age-related change in membrane progesterone receptors, which play an important role in progesterone and its derivative actions (Guennoun et al., 2015), has still to be determined.

Concerning androgen receptors, mRNA levels of AR have been reported to decrease in cerebral cortex and to increase in the hypothalamus of aged male mice (Munetomo et al., 2015). In contrast, a decrease in AR mRNA expression has been reported in the hypothalamic arcuate nucleus of the male rhesus macaques (Eghlidi et al., 2017).

## Mitochondrial Sexual Dimorphism in Brain Aging

The question of sex differences in normal aging goes beyond a fundamental scientific interest and is an essential step in medical research. The pharmacological evidences describing the enhancement of brain mitochondrial metabolism following systemic supplementation with sex steroids suggest that the age-induced decrease in sexual steroid production could contribute to brain mitochondrial decay (Chen et al., 2009b; Gaignard et al., 2017). It must be stressed however that almost all data about age-induced effects on mitochondrial RC or oxidative stress were obtained in male rodents. This feature is relatively common in experimental research (Zucker and Beery, 2010), but constitutes a serious lack for our thematic, as sex steroids influence mitochondrial function. The decrease of sex steroid hormones is an important turning point in the aging process in both sexes, but the reduction in estrogen blood levels during menopause is more abrupt than the reduction in testosterone levels during andropause. Thus, the consequences of aging on mitochondrial metabolism likely differ between women (or females) and men (or males). Surprisingly, this issue has been rarely addressed and especially in normal aging.

**Figure 4:**
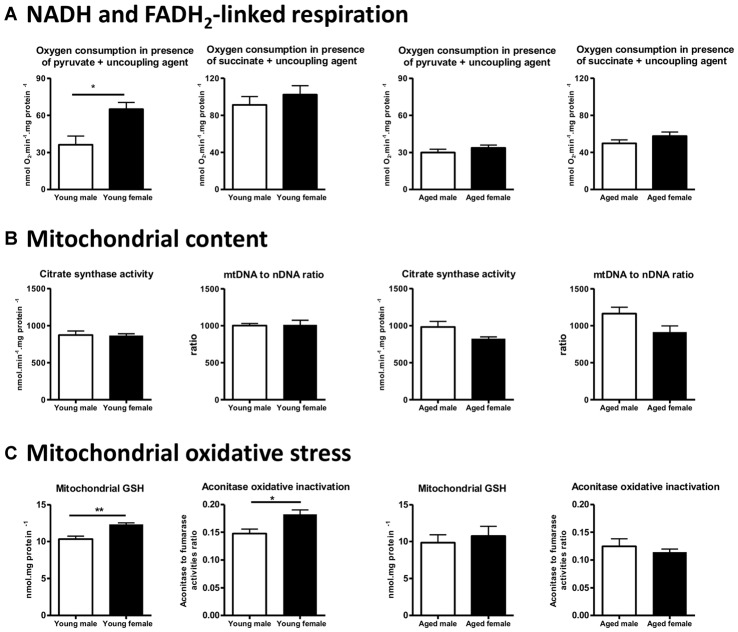
Sex differences in brain mitochondrial metabolism in young (3-month-old) and aged (20-month-old) male and female mice.** (A)** NADH-linked respiration rate (measured by the oxygen consumption in presence of pyruvate) was higher in young females (diestrus stage) than in young males and this sex difference disappeared in aged mice. **(B)** Mitochondrial content estimated by the citrate synthase activity and the mtDNA to nDNA ratio was not different between males and females in both young and aged mice. **(C)** Mitochondrial GSH pool was higher and oxidative inactivation of aconitase was lower in young females than in young males. These differences did not persist in aged mice. Data represent mean ± SEM of 5–6 mice. Statistical analysis: *t*-test. Significance: *p < 0.05; **p < 0.01. mtDNA, mitochondrial DNA; nDNA, nuclear DNA; GSH, reduced glutathione. Data from Gaignard et al. (2015).

We designed a specific study to determine whether brain mitochondrial metabolism is sexually dimorphic, in young and aged mice. We have measured the mitochondrial oxygen consumption and the activities of associated enzymes (the NADH-linked respiration, that depends on PDHc, TCA cycle enzymes and complexes I, III and IV activities and the FADH_2_-linked respiration that depends on complexes II, III and IV activities), the mitochondrial content (estimated by the citrate synthase activity and the mtDNA to nDNA ratio), the mitochondrial anti-oxidant protection (mitochondrial and total GSH pools) and the mitochondrial oxidative damages (oxidative inactivation of mitochondrial aconitase) in intact male and female mice at two ages: 3-month-old and 20-month-old. To standardize, the young adult females used were all in the diestrus stage and the old females were aged of 20 months to ensure that they were reproductively senescent. We showed that the NADH-linked respiration rate was higher in young females when compared to young males, and that it was related to a higher PDHc activity; the oxidative stress was lower in young females than in young males. By comparison, no significant difference was detected between 20-month-old male and female mice, neither in the respiration rate, the mitochondrial content nor in mitochondrial oxidative stress (Figure 4; Gaignard et al., 2015). Thus, the elevated mitochondrial metabolism observed in young females compared to young males disappears with aging.

By contrast, using Wistar rats, Guevara et al. (2009, 2011) reported that mitochondrial respiration (expressed per gram of tissue) was not different between 6-month-old males and females and between 24-month-old males and females. Oxidative damages were lower in young females when compared to males and this difference persisted in aged animals. The comparison between Guevara’s studies in Wistar rats and ours in C57BL/6 mice illustrates some essential methodological points that must be taken into account. First, discrepancy in the protocols used to measure oxygen consumption could explain why conclusions differ: the use of a high concentration of malate (associated with pyruvate) to start NADH-linked respiration may lead to a bypass of PDHc activity and may mask differences on it. Second, the choice of the unit expression is crucial: the complex IV activity, expressed per gram of tissue, was higher in aged female rats when compared to aged male rats but was not statistically different when expressed per milligram of mitochondrial proteins in Guevara’s study. These data suggest that mitochondrial content per cell or per gram of tissue varies according to sex and age. Guevara et al. (2009, 2011) reported that mitochondrial proteins to mtDNA ratio was higher in females than in males, young or aged. In our study, we evaluated mitochondrial content by two well-known markers (mtDNA to nDNA ratio and citrate synthase activity; Chretien et al., 1994; Medeiros, 2008), but we did not detect any difference between male and female mice either young or aged (Figure 4). It has been recently demonstrated that the brain expression of PGC1-α mRNA, the co-activator of NRF-1 and NRF-2, was lower in 22-month-old female mice when compared to male mice of the same age, but the impact on mitochondrial content was not evaluated (Zawada et al., 2015). Further studies, specially designed to analyze mitochondrial biogenesis by complementary methods, are necessary to determine whether mitochondrial content varies between males and females and the effect of aging. Third, it must be noted that rat and mouse mitochondrial metabolisms are not comparable. Studies revealed that, in males, ROS generation in the brain differed between Sprague-Dawley rats and C57BL/6 mice (Panov et al., 2007). Besides, in Wistar rats, young females produced less H_2_O_2_ than their male counterparts (Borrás et al., 2003), whereas the rate of anion superoxide generation was equivalent between males and females young C57BL/6 mice (Ali et al., 2006). Moreover, both brain steroid levels and their effects present substantial differences between mice and rat (Kellogg and Frye, 1999; Ebner et al., 2006; Liu et al., 2012; Porcu and Morrow, 2014).

**Figure 5:**
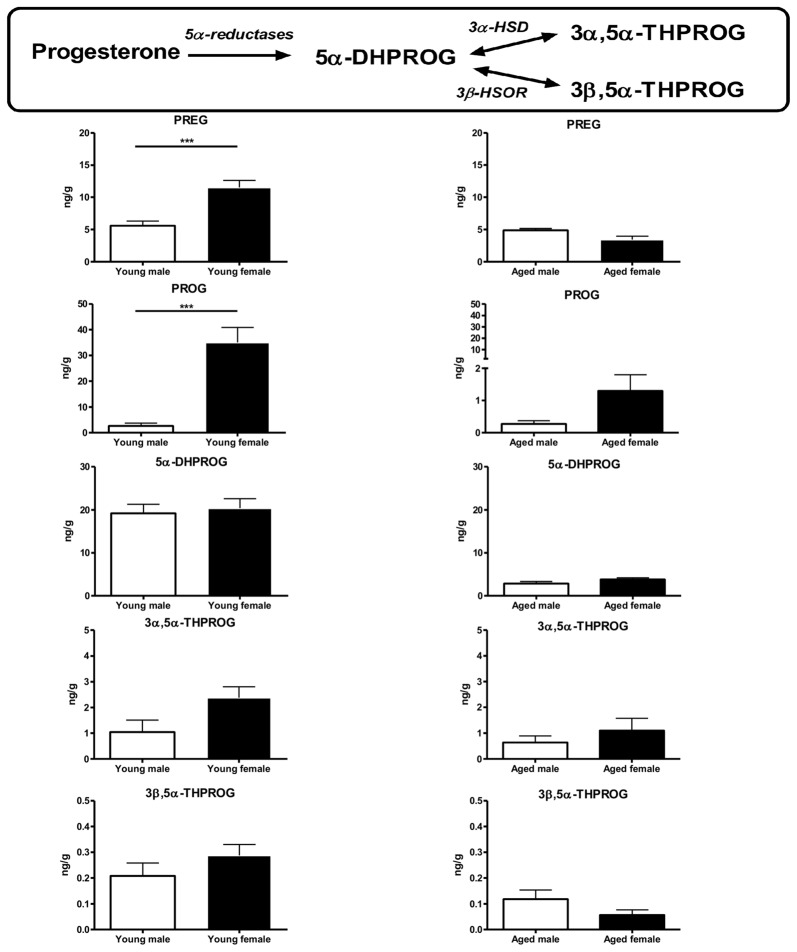
Brain levels of progesterone and its metabolites in young (3-month-old) and aged (20-month-old) male and female mice. Young females (diestrus stage) had higher brain levels of pregnenolone and progesterone when compared to young males. These differences disappeared in aged mice. The levels of 5α-reduced metabolites were not statistically different between sexes in young or aged mice. Data represent mean ± SEM of 4–6 mice. Statistical analysis: *t*-test. Significance: ***p < 0.001. 3α-HSD, 3α-hydroxysteroid dehydrogenase; 3β-HSOR, 3β-hydroxysteroid oxidoreductase; 5α-DHPROG, 5α-dihydroprogesterone; 3α,5α-THPROG, 3α,5α-tetrahydroprogesterone or allopregnanolone; 3β,5α-THPROG, 3β,5α-tetrahydroprogesterone or iso-allopregnanolone; PREG, pregnenolone; PROG, progesterone. Data from Gaignard et al. (2015).

The latter point is essential since we demonstrated that sex differences observed in brain mitochondrial metabolism are dependent on steroid levels. Thus, in our study, we performed gonadectomy in young adult male and female mice and we reported that the higher respiration rate and anti-oxidant protection in young female comparatively to young male mice were suppressed 3 weeks after ovariectomy. This finding strongly suggests a role of sex steroids in the male/female differences observed in young adults. By contrast, we have shown that three-week orchidectomy was without effect on male mitochondrial metabolism. Taken together, these results oriented us towards a major role played by the “ovarian steroids”, *i.e*., progesterone and 17β-estradiol, in the observed sex-difference. To test this hypothesis, we then have concomitantly analyzed mitochondrial function in one hemisphere and brain steroid levels in the contralateral hemisphere from the same animals using both male and female, young and aged mice. We showed that pregnenolone and progesterone levels were higher in young female mice (diestrus stage) when compared to young males. The 5α-reduced progesterone derivates levels (5α-dihydroprogesterone, 3α,5α-tetrahydroprogesterone and 3α,5β-tetrahydroprogesterone) were not statistically different between both sexes. In aged mice, pregnenolone and progesterone levels were lower than in young mice and the sex differences were no longer observed (Figure 5). Therefore, we postulated that the strong decrease of sex steroid levels and especially progesterone levels in aged female mice could diminish the “metabolic advantage” observed in young females (Gaignard et al., 2015).

Regarding the role of 17β-estradiol, its brain levels were too low to be measured by the accurate GC/MS method in our study. Nevertheless, Brinton (2008) has well described the role of a drop in 17β-estradiol in mitochondrial metabolism regulation. Based on pharmacological studies using exogenous 17β-estradiol administration to ovariectomized female rats, they showed that 17β-estradiol increased PDHc activity (that links glycolytic and oxidative glucose metabolisms), mitochondrial oxygen consumption and complex IV activity in brain (Nilsen et al., 2007; Irwin et al., 2008). They next postulated that the estrogenic impregnation during the reproductive period could enhance brain metabolism in young females by promoting the aerobic glycolysis. In women at the time of menopause, the reduction of ovarian hormones could lead to an hypo-metabolism that could predispose to neurodegenerative diseases (Brinton, 2008). Experimental studies by the same team then confirmed that PDHc activity, mitochondrial oxygen consumption and complex IV activity declined during reproductive senescence in female mice and, using the model of ovariectomized females, that these decreases were reversed by 17β-estradiol substitution (Yao et al., 2010, 2012; Irwin et al., 2011). Interestingly, in our work comparing males and females, we also showed that young females had higher PDHc activity than young males (Gaignard et al., 2015). Recently, a proteomic and functional study concerning the evolution of brain mitochondrial metabolism during aging performed in males, has completed the picture. Stauch et al. (2015) reported that the expression of glycolytic enzymes was higher in mitochondria isolated from brains of 12-month-old and 24-month-old C57BL/6 male mice when compared to those isolated from 5-month-old male mice. By contrast, the TCA cycle enzymes and RC complexes expressions declined from 12 months to 24 months. Functional analysis revealed that oxygen consumption appeared unchanged with aging in males. In light of these data, the role played by the cytoplasmic glycolytic enzymes and PDHc in the sexually dimorphic effects of age on mitochondrial metabolism has to be more considered.

It seems that the mitochondrial effects of sex steroids in endogenous conditions could be more important in the brain than in the other tissues. In fact, no male/female differences were observed in energetic function of mitochondria isolated from heart, skeletal muscle and liver of C57BL/6 mice (Sanz et al., 2007; Khalifa et al., 2017). Besides, the sex difference on PGC1-α mRNA expression seen in brain of aged mice was not retrieved neither in the liver nor in the kidney (Zawada et al., 2015). Detailed studies in several species are now necessary to better determine the extent and the mechanisms of mitochondrial sexual dimorphism during aging.

The two strains of the senescence-accelerated mice (SAM), one prone to accelerated senescence (SAMP) and one resistant (SAMR), are attractive models for aging research. Data about brain mitochondrial metabolism in either SAMR1 or SAMP8 10-month-old male and female mice were reported by two studies. These studies were designed to analyze melatonin effects on mitochondria but the comparison between male and female control groups may provide information. In SAMR1 mice (the “young” model), no sex-related difference was detected in brain mitochondria lipid peroxidation, GSH/GSSG ratio and RC complexes activities. In contrast, in the SAMP8 mice (the “aged” model), females presented a higher lipid peroxidation, a lower GSH/GSSG ratio and lower complexes I and III activities when compared to their male counterparts (Carretero et al., 2009; Escames et al., 2013). In brief, mitochondrial sexual dimorphism was present in the aged model but not in the young model. The discrepancy between these results and ours is probably due to the particular endocrine profile of the SAM mice, making them unsuitable for studying endogenous steroid influences (Yuan et al., 2005). Nevertheless, the classic rodent models are also not entirely satisfactory, since the decrease in circulating sex steroids during reproductive senescence (the “menopause” in rodents) is primarily caused by the decrease in hypothalamic pituitary axis signal and not by the decline of follicles supply, like in women (Yin and Gore, 2006).

Dedicated studies exploring sexual dimorphism in aging-induced variations in brain metabolism are still rare in humans and particularly in cognitively intact individuals. The studies previously cited about the decrease of RC complexes activities or quantities, included men and women but did not specifically address the question of sex difference (Ojaimi et al., 1999; Cottrell et al., 2001; Venkateshappa et al., 2012). At the transcriptome level, two gene expression studies revealed that responses in aging are sexually dimorphic in human brain, and particularly concerning metabolic regulation (Berchtold et al., 2008; Guebel and Torres, 2016). In an analysis of age and sex effects on hippocampus transcriptome in men and women, 500 probes related to mitochondrial function were identified, and the age-induced effects were different according to the sex for one third of them. Among the major features, the expression of genes involved in mitochondrial fission (i.e., the fragmentation of one mitochondrion to generate other mitochondria) was higher in older women than in older men, suggesting possible sex differences in brain mitochondrial network (Guebel and Torres, 2016). The biological significance of the strong age and sex interactions observed in the expression of *HMGCS2*, a gene encoding the 3-hydroxy-3-methylglutaryl-CoA synthase involved in hepatic ketogenesis but with splice variants expressed in brain (Puisac et al., 2012) and in the expression of *UCP3*, a gene encoding uncoupling protein and expressed at low level in brain (Alán et al., 2009), has to be determined. In another study Berchtold et al. (2008) showed that age-related changes in gene expression were more accentuated in men than in women. Especially, the expression of genes involved in energy production (electron transfer, ATP production) was more decreased during aging in men than in women in several regions of the brain. These data run rather counter the Brinton’s and Stauch’s teams works in rats showing a hypo-metabolism during female aging but not during male aging (see above).

## Mitochondrial Sexual Dimorphism in Age-Related Neurodegenerative Diseases

The influence of sex on various pathologies, and specially on neurodegenerative diseases, has become an extensive field of investigations, and mitochondrial metabolism seems to play a key role (Ventura-Clapier et al., 2017). We will focus on mitochondrial bioenergetics and oxidative stress regulations and the influences of sex steroids on it in the two major age-related neuronal diseases: AD and Parkinson’s disease (PD).

### Alzheimer’s Disease

AD is the most common cause of dementia in aged people and is characterized by a gradual cognitive decline. The histopathological features of AD are: extracellular plaques constituted by accumulation of β-amyloid peptides (Aβ); and intraneuronal inclusions of neurofibrillary tangles composed of hyperphosphorylated forms of tau, a microtubule-associated protein. The lesions predominate in cortex and hippocampus (Grundke-Iqbal et al., 1987; Hardy and Higgins, 1992).

Among the multiple and intricate physiopathological mechanisms of AD, mitochondria play a pivotal role by controlling calcium homeostasis, intrinsic apoptosis but also energy production and oxidative stress. Deficits in the oxidative phosphorylation system have been described in AD patients by Sims et al. (1987) 30 years ago and confirmed by numerous studies so far (for review, Johri and Beal, 2012; Wang and Brinton, 2016). Beyond the RC failure, a decline in PDHc and in some TCA cycle enzymes were also described in AD human brains as well as a reduction in cerebral glucose utilization (Ishii et al., 1997; Blass et al., 2000). The decrease in energy production is associated with an increase in oxidative stress (for review, Wang and Brinton, 2016). More recently, dysregulation in mitochondrial dynamics has also been described in AD (Zorzano and Claret, 2015).

Mitochondrial dysfunction has been proposed to be either as a consequence or a cause of Aβ and hyperphosphorylated tau accumulations. In the “amyloid cascade hypothesis”, the Aβ accumulation causes mitochondrial toxicity, which leads to an impairment of mitochondrial energy (Hardy and Higgins, 1992). By contrast, in the “mitochondrial cascade hypothesis” elaborated by Swerdlow and Khan, the mitochondrial dysfunction initiates Aβ accumulation (Swerdlow and Khan, 2004). According to this theory, the combination of inherited mutations on mtDNA (called mtDNA haplogroup) determines the baseline of mitochondrial function for each individual. The age-associated mitochondrial decline rate is then influenced by genetic and environmental factors. If the mitochondrial decline surpasses a threshold, it results in less energy synthesis, increased ROS production and disrupted calcium homeostasis. This mitochondrial failure would next perturb the control of Aβ production and tau phosphorylation. For example, it has been proposed that excess of mitochondrial oxidative stress and impaired mitophagy (the selective degradation of mitochondria) could disturb amyloidogenic processing and trigger hyperphosphorylation of tau (Melov et al., 2007; Kerr et al., 2017). The accumulation of Aβ and hyperphosphorylated tau then exacerbates mitochondrial failure by a vicious circle process (Swerdlow and Khan, 2004; Swerdlow et al., 2014).

Various epidemiological studies showed that AD affects more aged women than aged men; almost two thirds of the individuals diagnosed are women. The higher incidence of AD cases in women compared to men could be attributed to several mechanisms like the longer lifespan of women and the frequency of associated co-morbidities promoting AD development. However, it was demonstrated that gender is an independent risk factor for AD, which could suggest an influence of chromosomal sex (XX or XY) and/or of sex steroid hormones (Mielke et al., 2014). Moreover, the evolution of the pathogenic hallmarks in AD animal models is also sexually dimorphic (for review, Grimm et al., 2016b). Probably because AD is highly multifactorial, epidemiological data failed to clearly support the hypothesis of sex steroid hormones influence. Several studies reported an increased risk of AD with early menopause, however no significant correlation between the age of menopause and AD incidence was established (Henderson and Brinton, 2010; Yao and Brinton, 2012). Nevertheless, pharmacological evidence in preclinical studies clearly indicates a beneficial effect of estrogen supplementation (Barron and Pike, 2012). Moreover, in women, the protective effects of hormone replacement therapy (HRT) were reported by several observational studies. However, these effects were not confirmed by interventional clinical trials, in particular the Women’s Health Initiative Memory Study (Hogervorst et al., 2009). The age of HRT initiation seems crucial to explain this discrepancy and introducing HRT early in the disease progress could be more beneficial (Chen et al., 2006; Henderson and Brinton, 2010). Associated risk factors such as mtDNA haplogroup or apolipoprotein E (ApoE) genotype should also be taken into account to better evaluate the role of endogenous steroids on AD incidence and protection and on the eventual benefit of HRT. Thus, some mtDNA haplogroups were found to be associated with increased or decreased risk of AD, but only in women or in contrary only in men (Wang and Brinton, 2016). For example, carrying the allele E4 of the gene encoding the ApoE is an important risk factor for AD because it could promote plaque aggregation and alter neuronal membrane regeneration and this genotype is also associated with a reduced efficacy of HRT (Thornton et al., 2011; Depypere et al., 2016; Wang and Brinton, 2016).

Based on the central position of mitochondria in AD pathogenesis and on the influence of sex on brain mitochondria function, it could be hypothesized that mitochondrial energetic metabolism and oxidative stress regulation are involved in AD sex bias. This hypothesis was explored in the model of the triple transgenic-AD mice (3xTg-AD mice) that develop both Aβ plaques and tangles. Coskun et al. (2012) showed that the evolution of brain mitochondrial respiration, complexes I and IV activities in 3xTg-AD mice during aging was different according to the sex: male 3xTg-AD mice presented lower mitochondrial respiration and complexes I and IV activities than age-matched control male mice as early as 1 month of age but these activities did not decline throughout the life (between 1 and 24 months of age). By contrast, young female 3xTg-AD mice had the same rate of mitochondrial respiration and same activities of complexes I and IV than age-matched control female mice but these functions significantly declined with aging. Therefore, old female 3xTg-AD mice exhibited lower mitochondrial functions when compared to age- and sex-matched controls (Coskun et al., 2012). The team of Brinton also showed that brain mitochondrial function (mitochondrial respiration and complex IV activity) decreased since the age of 9 months in female 3xTg-AD mice. In parallel with RC decline, the mitochondrial lipid peroxidation and the free radical leak strongly increased in aged 3xTg-AD female mice (Yao et al., 2009, 2010). The link between low ovarian steroids levels and exacerbated mitochondrial dysfunction was also described in ApoE4 mice, another model of AD mice. The detrimental effect of ApoE4 genotype vs. ApoE3 genotype on synaptic mitochondrial proteome was greater in females than in males. Yet, ApoE4 females presented low cortical 17β-estradiol levels than the ApoE3 control female mice (Shi et al., 2014). In *ex vivo* experimentations using mitochondria isolated from brain of wild-type (WT) Wistar rats incubated with Aβ, it has also been shown that the oxidative stress response was different between young and aged females. Peroxide production and cytochrome c release were strongly enhanced after Aβ addition to mitochondria isolated from young males or from aged females. By contrast, mitochondria isolated from young females were protected against the toxicity of Aβ (Viña et al., 2007).

In addition to RC dysfunction and subsequent ROS production, a decrease of PDHc activity was described in 3xTg-AD female mice since the age of 9 months. Interestingly, PDH subunit E1α expression was decreased as early as 3 months of age, but the PDHc activity was not modified, probably thanks to compensatory post-translational modifications (Yao et al., 2009, 2010). In addition, the activities of the hydroxyacyl-co enzyme A deshydrogenase (HADHA, an enzyme involved in the fatty acid oxidation) and of the 3-oxoacid-CoA transferase 1 (SCOT, an enzyme involved in the ketolysis) were increased in 3-month-old 3xTg-AD female mice when compared to WT mice. The authors suggest that the activation of the fatty acid oxidation (that produces acetyl-coA which is then converted into soluble ketone bodies) and the ketolysis pathway (that reconverts ketone bodies into acetyl-coA) is a way to compensate the reduction in the pool of acetyl-coA due to the decrease of PDHc activity. During reproductive senescence, the expressions of SCOT and HADHA increased in WT aged females in parallel with the PDHc decrease. By contrast, in aged 3xTg-AD female mice, the SCOT expression did not increase; as a consequence, the utilization of ketone bodies as alternative fuel could be limited leading to aggravation of the energetic deficit (Yao et al., 2009, 2010). Recently, the same team has proposed a mechanism for the switch of energetic fuel during normal reproductive senescence: the mitochondrial decline could enhance peroxide production that activates the cytosolic phospholipase A2-sphingomyelinase pathway. The lipids liberated from myelin breakdown could be a source for the fatty acid oxidation by the astrocytes. The astrocytes then provide ketone bodies to the neurons in order to furnish acetyl-coA for the TCA cycle. In the context of AD, the glucose metabolism is decreased right from the prodromal phase and the activation of this adaptive pathway could be a cause of early white matter degeneration (Yao and Brinton, 2012; Klosinski et al., 2015).

All these findings strongly orient toward an important influence of ovarian steroids on the evolution of mitochondrial AD-induced disorders: the impregnation by estrogens and progestagens could protect young females during the reproductive period but the strong drop at the time of menopause could precipitate the mitochondrial decline and disturb the brain homeostasis of Aβ production and tau phosphorylation. Experimental pharmacological studies principally focused on the role of estrogens and showed that 17β-estradiol is protective against the oxidative stress increase and the oxidative phosphorylation decrease observed in AD models (Viña et al., 2007; Yao and Brinton, 2012). The properties of the two other groups of sex steroids, progestagens and androgens, have been less tested (see review, Grimm et al., 2016b). Recently, Grimm et al. (2016a) performed an exhaustive study on the effects of sex steroids on mitochondrial function in two cellular models of AD, one mimicked Aβ accumulation (neuroblastoma cells transfected with the human amyloid precursor protein APP) and the other mimicked tau hyperphosphorylation (neuroblastoma cells transfected with mutant tau P301L). In this work, the effects of progesterone, 17β-estradiol, estrone, testosterone or 3α-androstanediol on mitochondrial ATP level, membrane potential and respiration rate were investigated. This study revealed very interesting findings: progesterone and 17β-estradiol were the most effective steroids to alleviate mitochondrial energetic failure in P301L cells, whereas testosterone was the most effective in APP cells. Therefore, young males could be better protected against Aβ-induced mitochondrial alterations; whereas the ovarian steroids could prevent abnormal tau-induced mitochondrial alterations in young females (Grimm et al., 2016a). One can note that these assays were performed in neuroblastoma cells and should be confirmed in other brain cells. Indeed, in embryonic rat hippocampi from WT rats, it has been reported that progesterone failed to protect neurons against mitochondrial toxins, whereas 17β-estradiol was protective (Yao et al., 2011). However, the Grimm’s study conclusions are consistent with an *in vivo* study in 3xTg-AD male mice showing that orchidectomy increased Aβ accumulation more than tau hyperphosphorylation in several brain regions including hippocampus (Rosario et al., 2010). Moreover, dihydrotestosterone (DHT) administration prevented Aβ accumulation but not tau hyperphosphorylation. In contrast to testosterone, DHT is not metabolized to 17β-estradiol by aromatase. The regulation of Aβ accumulation is therefore well mediated by a specific androgenic effect (Rosario et al., 2010). Thus, it has been suggested that the gradual decrease in testosterone during andropause may participate in AD pathogenesis in aged men (Grimm et al., 2016b).

Besides oxidative phosphorylation and oxidative stress, Aβ and hyperphosphorylated tau also alter mitochondrial dynamics (the balance between fusion and fission; Wang et al., 2008; DuBoff et al., 2012). Interestingly, the regulation of mitochondrial dynamics by sex steroids has been described as sexually dimorphic. Thus, 17β-estradiol or progesterone addition increased the expression of both mitochondrial fusion and fission genes in cortical astrocytes from WT female mice. In contrast, in WT male mice cortical astrocytes, 17β-estradiol enhanced the expression of fission genes and of one fusion gene (*MFN2*) and decreased the expression of the second fusion gene *(MFN1)*; while progesterone decreased both fission and fusion gene expressions (Arnold et al., 2008). In AD models, the effects of sex steroids on mitochondrial network have not been described yet.

Like for normal aging, exploring human brain mitochondrial metabolism in AD patients in order to objectivize putative sexual dimorphism is obviously challenging. Thanks to the progress of proteomics, a recent study described sex-specific changes in proteome of mitochondria from temporal lobes of patients suffering from AD with cerebrovascular disease. Some subunits of complexes I, III and IV were down-regulated and subunits of complex V were dysregulated in women when compared to men (Gallart-Palau et al., 2016). These findings are in agreement with the data obtained in rodents (Yao et al., 2009, 2010; Coskun et al., 2012). Surprisingly, in contrast of what was reported in rodents, the E1β subunit of PDHc was down-regulated in humans, but only in men (Gallart-Palau et al., 2016). Additional studies in other brain regions and in other forms of AD are now necessary to extend the knowledge about sex-specific mitochondrial alterations in AD and their consequences for potential new sex-based therapies.

Together, the recent findings highlight that in addition to sex differences in mitochondrial function, the effects of sex steroids on mitochondria could differ between men and women. Consequently, the development of new sex-specific therapies seems to be a promising way in AD treatment.

### Parkinson’s Disease

PD is the second most common age-related neurodegenerative disorder and is defined by a triad of motor symptoms associating bradykinesia, rigidity and resting tremor. Non-motor symptoms such as inaugural depression or delayed impaired cognition are also frequent. PD pathological hallmarks are the degeneration of dopaminergic neurons in the substantia nigra and subsequent striatal dopamine loss and the presence of intra-neuronal protein inclusions of aggregated α-synuclein proteins called Lewy bodies in the brainstem and the neocortex (Lees et al., 2009).

The involvement of mitochondrial dysfunction in PD pathogenesis is supported by evidence accumulated over the last several decades. The most described mitochondrial dysfunction is the decrease of complex I activity. Deficiencies in complex I were reported in substantia nigra, platelets and skeletal muscle of patients with PD. Moreover, when complex I is selectively inhibited by rotenone, 1-methyl-4-phenyl-1,2,3,6-tetrahydropyridine (MPTP) or its metabolized form N-methyl-4-phenylpyridinium ion (MPP^+^), the symptoms and the neuropathological features in corresponding animal models are similar to those observed in humans (for review, Johri and Beal, 2012; Chaturvedi and Flint Beal, 2013). Like in AD, an association between PD cases and some mtDNA haplogroups has been reported, suggesting that some patterns of inherited mtDNA mutations could promote mitochondrial dysfunction. Moreover, complex I deficiency was observed in “cybrids”, a technic that consists in introducing mitochondria from PD patients in a healthy nuclear background (Chaturvedi and Flint Beal, 2013). However, it seems that down-regulation of RC subunits is not only due to a reduction of mtDNA-encoded subunits expression. Recently, a meta-analysis of several genome-wide expression studies revealed that the expression of nuclear genes encoding complex I subunits but also complexes II, III, IV and V subunits were down-regulated in dopaminergic neurons of patients with PD. The authors underline that most of these genes are under the control of PGC1-α, a major nuclear regulator of mitochondrial biogenesis (Zheng et al., 2010). Upstream the RC, a decrease of αKGDH activity, a TCA cycle enzyme, was also described in PD patients (Gibson et al., 2003). The rare familial forms of PD, due to mutations in nuclear genes, also strongly suggest that mitochondrial dysfunction is pivotal in PD pathogenesis. For example, mutations that affect the protein Parkin (the phosphatase and tensin homolog induced kinase 1, PINK1) lead to impairments in mitophagy or in mitochondrial dynamics (Chaturvedi and Flint Beal, 2013). Finally, the deficiency of complex I seems to be the most ostentatious but not the only mitochondrial alteration in PD. The precise process remains elusive but the energy production failure and the subsequent oxidative stress are certainly involved in dopaminergic neuron degeneration.

Sex ratio in PD is opposed to the one observed in AD: the men are twice more affected than women. The clinical profile and the response to treatment are also marked by sex differences. This trend is reproduced in animal models: the administration of 6-hydroxydopamine (6-OHDA, a toxic hydroxylated analog of dopamine) induces more loss of dopaminergic neurons in males than in females (for review, Gillies et al., 2014). The ability of the sex-determining region Y (SRY) protein to regulate dopamine synthesis suggests that chromosomal sex is involved in PD sexual dimorphism (Czech et al., 2012). Nevertheless, ovarian hormones are most probably involved too, as proved by epidemiological data like the increased risk of PD in women after bilateral oophorectomy (Rocca et al., 2008) or by some data suggesting a protective effect of early HRT in women (Bourque et al., 2009; Gillies et al., 2014). Importantly, estrogens seem to be protective only in females. In animal experiments with 6-OHDA administration, it has been shown that ovariectomy enhanced the loss of striatal dopamine, whereas orchidectomy limited it and that 17β-estradiol administration reduced striatal lesions in females but increased it in males. It has also been hypothesized that androgens could modulate toxin-induced PD alterations, since men are more affected than women (Gillies et al., 2014). Yet, no study reported a specific androgenic effect (Ekue et al., 2002; Bourque et al., 2009; Gillies et al., 2014).

The mechanisms underlying beneficial effects of estrogens and the resulting PD sex bias are increasingly better understood. It has been demonstrated that estrogens influence the activation of apoptotic pathways in dopaminergic neurons, the dopamine transporter, the brain renin-angiotensin system and the glial inflammation (Morale et al., 2006; Bourque et al., 2009; Gillies et al., 2014; Labandeira-Garcia et al., 2016). Considering the sex influence on mitochondrial metabolism during normal aging and the key role of mitochondria in PD disease, it is essential to investigate male/female differences in mitochondrial functions in PD models. Few studies addressed this issue so far. In 3-month-old Swiss albino mice, neither acute nor chronic administration of MPTP triggered a decrease of complex I activity in striatum or mesencephalon from females; whereas males exhibited a strong deficit. Pretreatment of females with an estrogen receptor antagonist (ICI 182, 170) abolished the sex difference (Kenchappa et al., 2004). This study also reported that intact young females presented a higher glutaredoxin 1 (Grx1) activity than males and that this up-regulation was also mediated by estrogens. Grx1 allows the reactivation of proteins oxidatively inactivated by S-glutathionylation, like some complex I subunits. The high Grx1 activity in females could protect complex I against oxidative stress consequences (Kenchappa et al., 2004). It would be interesting to test if the same phenomenon occurs with αKGDH activity. Indeed, αKGDH is prone to inactivation by S-glutathionylation and its activity is rate-limiting for TCA cycle functioning in brain (Tretter and Adam-Vizi, 2005; Bénit et al., 2014; Ribas et al., 2014). The regulation of RC genes expression is also sexually dimorphic (Misiak et al., 2010). Thus, in primary mesencephalic neuron cultures from male or female fetus mice, it has been shown that the addition of 6-OHDPA induced a decrease of the expressions of mtDNA- and nDNA-encoded RC subunits and that this decrease was more pronounced in male neurons than in female neurons for some mtDNA-encoded subunits. The 6-OHDA addition also triggered a decrease of ATP levels and an increase of ROS levels and these effects were more important in males. Furthermore, the administration of 17β-estradiol counteracted the increase in ROS levels but not the decrease in ATP levels in both sexes (Misiak et al., 2010). Sex-specific toxin-induced mitochondrial alterations were reported in glial cells as well. The addition of MPP^+^ to mesencephalic astrocytes increased the expression of the subunits 1 and 2 of the complex IV, decreased ATP levels and increased peroxide production, but only in cells from male mice pups; whereas no or attenuated effects were observed in cells from female mice pups (Sundar Boyalla et al., 2011). Another study reported that the striatal astrocytes from male mice were more sensitive to MPP^+^-induced toxicity than striatal astrocytes from female mice. The higher levels of paraoxonase 2 (PON2), a 17β-estradiol inducible antioxidant lactonase, in females could participate to the protection (Giordano et al., 2013). Together, these results suggest that ovarian steroid impregnation of young females is protective against PD-induced mitochondrial alterations.

**Figure 6:**
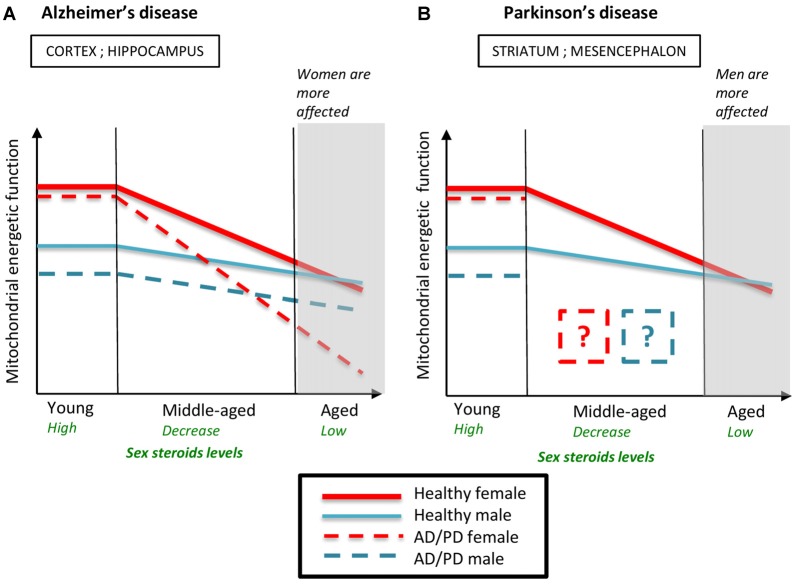
Schematic representation of the evolution of mitochondrial energetic function in males and females during normal and pathological aging. Young healthy females have higher mitochondrial energetic function than young healthy males. The decline of ovarian steroids during reproductive senescence accentuates the age-induced mitochondrial decline. Mitochondrial energetic function is similar between aged males and females. **(A)** In Alzheimer’s disease (AD) models, young females are more protected against AD-induced mitochondrial failure than young males but the drop of ovarian steroids levels during reproductive senescence strongly precipitates mitochondrial impairment. Therefore, mitochondrial function is more altered in AD aged females than in AD aged males, which is consistent with the sex ratio observed in AD. **(B)** In Parkinson’s disease (PD) models, young females are also more protected than young males. Data on mitochondrial function in middle-aged and aged PD animals are lacking. AD, Alzheimer’s disease; PD, Parkinson’s disease. Data from Kenchappa et al. (2004), Yao et al. (2009, 2010), Misiak et al. (2010), Sundar Boyalla et al. (2011), Coskun et al. (2012) and Gaignard et al. (2015).

By comparing the mitochondrial mechanisms implied in AD and PD pathogenesis, some similarities appear: the energy production failure and the increase of oxidative stress are central in the two diseases; ovarian steroids impregnation in young females is protective (Figure 6). In AD, previously cited studies thoroughly suggest that the drop of ovarian steroids leads to a hypo-metabolic state that could precipitate the mitochondrial failure and disturb Aβ and tau homeostasis (Yao et al., 2009, 2010; Coskun et al., 2012). Whether a similar mechanism occurs in PD is not yet determined and is not so obvious because some molecular mechanisms described in brain regions involved in AD or PD pathogenesis could be missing or different in other brain regions. For example, the protective effect of high levels of PON2 in females that was described in striatal astrocytes could not be effective in the cortex and the hippocampus, since PON2 is expressed at low level in these brain regions (Giordano et al., 2011, 2013). After MPP^+^ addition, the ATP levels were more decreased in mesencephalic astrocytes from males than from females, as evoked. Conversely, in cortical astrocytes, the MPP^+^ addition decreased more ATP levels in cells from females than in cells from males (Sundar Boyalla et al., 2011). Therefore, further explorations should now investigate whether the protection observed in PD young females is abolished or even reversed in PD aged females after the drop of ovarian steroids. In addition, data about sex-specific mitochondrial metabolism differences in human patients are still lacking and should be undertaken to complete the puzzle of PD sex bias and to design the most appropriate therapy according to the sex and the hormonal status.

## Conclusion

Mitochondria are in charge of energy production, regulate oxidative stress and are the site of the first steps of steroidogenesis. In addition, they are target of sex steroids. As the nervous system has a high metabolic rate and a low capacity of energy storage, dysfunction of brain mitochondria has devastating consequences. Several indications show that brain mitochondrial functions decline with age and that sex steroid loss is involved in the observed dysregulation of mitochondrial functions. Furthermore, sex differences in brain mitochondrial functions may explain, at least partially, the influence of sex steroids on neurodegenerative diseases such as AD and PD. These data describing the complex relationships between age, sex steroids and mitochondrial function should be taken into account when designing therapies for successful aging of both men and women. Investigating the mechanisms and optimizing the strategies of steroid supplementation, will help in better developing sex-specific cerebroprotective approaches.

## Author Contributions

PG and RG conceived, designed and drafted the manuscript. All authors revised critically and approved the final version.

## Conflict of Interest Statement

The authors declare that the research was conducted in the absence of any commercial or financial relationships that could be construed as a potential conflict of interest.
